# Evaluation of toxic effects of benzophenone on histopathology of *Labeo rohita*

**DOI:** 10.1016/j.toxrep.2025.101914

**Published:** 2025-01-14

**Authors:** Maham Riaz, Sajid Abdullah, Mina Jamil, Amna Rasheed, Urwah Sheikh, Maham Fatima, Nimra Umer, Kaynat Aslam

**Affiliations:** Department of Zoology, Wildlife & Fisheries, University of Agriculture, Faisalabad, Pakistan

**Keywords:** Histopathology, Benzophenone, *Labeo rohita*, Ultraviolet filters, Chronic toxicity

## Abstract

Benzophenone (BP) is an organic ultraviolet (UV) filter widely used in sunscreens and personal care products. This compound enters aquatic ecosystems due to industrialization, wastewater treatment plants (WWTPs), and domestic effluents, poses serious threats to aquatic organisms, and is considered an emerging pollutant. This laboratory-based study assessed the 96-hour (h) median lethal concentrations (LC_50_) and sub-lethal effects of BP on the histology of the gills and muscles of *Labeo rohita*. Fish fingerlings of the same weight (48 ± 2 g) and length (5 ± 2 in.) were exposed to gradually increasing concentrations of BP (100 µg/L to 1000 µg/L) and their 96-h LC_50_ was determined as 612.822 ± 37.38 µg/L. To determine the sub-lethal effects, the fish were exposed to 1/5th of the 96-h LC_50_ of BP for 35 days (d) to investigate organ-specific responses. The results indicated significant damage to the exposed organs and showed damage in pillar cells and intraluminal debris in gill mucous cells. Moreover, fragmentation of intact muscle structures, intraluminal debris, and vascular necrosis were observed in exposed muscles*.* In conclusion, these results confirmed the histopathological changes in the gills and muscles of *L. rohita* caused by BP exposure, thereby confirming its risk to aquatic life.

## Graphical Abstract

**Figure:**
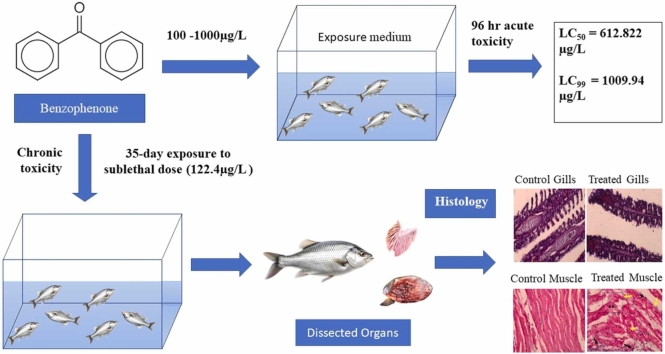

## 1 Introduction

Aquatic environments become polluted when undesirable substances enter water bodies and alter their quality [4], and are considered the final destination for contaminants produced naturally and through human activity. [16]. Aquatic environments are susceptible to industrial, household and urban waste pollution [8]. These contaminants threaten the health of aquatic organisms [14] and ecosystems [29].

Benzophenone (BP) is a chemical compound that is added to sunscreens, cosmetics, and personal care products for sun protection and is widely used in ultraviolet (UV) filters worldwide [11]. This pollutant may reach aquatic ecosystems directly via industrial effluent due to inadequate removal, surface runoff, or activities such as swimming, or indirectly via wastewater treatment plants (WWTPs) after bathing [17]. WWTPs are not designed to remove them effectively; therefore, BP UV filters can accumulate in WWTPs effluents [28]. The extensive use of BP UV filters and their inefficient removal from wastewater have increased their concentration in water, raising concerns regarding their potential toxicity and health effects on aquatic environments. Maximum levels of BP UV filters are reported in the river (44 micrograms per liter), seawater (34.3 micrograms per liter), waste-water influent (10.4 micrograms per liter), swimming pool (4.5 micrograms per liter), tap water (0.45 micrograms per liter), lake (0.2 micrograms per liter), and groundwater (0.034 micrograms per liter) [21].

Most toxicological studies of BP UV filters in aquatic fish species have focused on their endocrine disruptive ability and impact on reproduction [7], [21]. Additionally, oxidative stress and histopathological changes in different organs of the fish body, such as the liver and gills, have been reported [39], [40]. Moreover, social behavior in Male Siamese fighting fish (*Betta splendens)*, immune and metabolic systems in zebrafish (*Danio rerio),* and clown anemone fish are also affected [31], [46]. Estrogenic effects, neurotoxicity, genotoxicity, developmental toxicity, and blood cell alterations have also been reported in embryonic and adult zebrafish and the rare minnow (*Gobiocypris rarus*) [10], [38], [44], [23], [42]. The European Union has prohibited the sale of products containing BP since November 20, 2023, due to its carcinogenic nature (CLP Regulation 1272/2008).

Fish species are excellent models for monitoring the presence of pollutants in aquatic environments [41] because they are susceptible to small amounts of contaminants within water bodies, are abundant, and are present in aquatic environments [1]. Histopathological indicators are frequently used in fish health research because they provide information on the chronic and sublethal effects of contaminants on organs as well as for evaluating fish stress. They are frequently used in studies on contaminated aquatic environments because they are reliable indicators of disrupted or contaminated environments. The most commonly used histopathological indicators for evaluating fish health are liver, kidneys, gills, and skin [26].

*Labeo rohita* is one of the most commonly cultured fish species worldwide, particularly in South Asia, and is widely used in research [12]**.** This freshwater fish plays a critical role in the ecological balance of aquatic environments by assisting in nutrient cycling and trophic interactions [15]. Several studies have evaluated the toxicity of BP in fish gills [24], [2], [40]. However, there is insufficient literature available on the evaluation of BP toxicity in fish muscle histology. We hypothesized that BP exposure causes histological damage to the muscles and gills of *L. rohita*; however, this theory requires further investigation.

## 2 Materials and methods

### 2.1 BP and fish acclimatization

This study was conducted at the Toxicological Research Lab, Department of Zoology, Wildlife and Fisheries, University of Agriculture, Faisalabad, Pakistan. BP (CAS # 119–61–9, 99 % purity; Sigma-Aldrich Louise, MO USA) was dissolved in 0.1 % dimethyl sulfoxide (DMSO) (CAS # 67–68–5) solvent to prepare a 100 mg/L stock solution, which was stored at −20 °C. Serial dilutions of the stock solution were made to prepare the working solutions according to the requirements.

Fingerlings of *L. rohita with* the same weight (48 ± 2 g) and length (5 ± 2 in.) were purchased from a local hatchery. Before the experiment, the fish were acclimatized for 10 days (d) in a static tank (70 L) containing reconstituted aerated tap water and fed commercial pelleted feed twice daily. During acclimatization, the water quality parameters were maintained (Table 1). The university’s ethical board approved the experimental protocol (CEE Council 86/609).

**Table 1:** The physicochemical parameters (Mean ± SD) of water maintained during the acclimatization period, LC_50_ determination, and sublethal exposure*.* The physiochemical parameters of water maintained during the acclimatization period, LC_50_ determination, and sub-lethal exposure to BP. The parameters were recorded using different analytical methods. The means ± Standard Deviation (SD) of these parameters were recorded.

**Parameters**	**Acclimatization period**	**LC**_**50**_**determination**	**Sublethal exposure**	**Analytical Method**
Temperature (°C)	30.01 ± 0.23	30.23 ± 0.46	28.98 ± 0.39	Temperature meter
pH	7.11 ± 0.15	7.48 ± 0.28	7.96 ± 0.30	pH meter
Total Hardness (mg/L)	248.51 ± 3.86	249.49 ± 4.96	249.67 ± 4.01	Titration method
Carbon dioxide (mg/L)	0.69 ± 0.13	0.76 ± 0.15	0.81 ± 0.18	Titration method
Magnesium (mg/L)	47.70 ± 0.78	48.83 ± 0.90	48.57 ± 0.85	Titration method
Calcium (mg/L)	23.78 ± 1.38	22.96 ± 1.40	23.01 ± 1.41	Titration method
DO (mg/L)	5.75 ± 0.27	5.66 ± 0.23	5.98 ± 0.25	DO meter

DO= dissolved oxygen

### 2.2 Experimental design

The experiment was performed in two phases: determination of the 96-hour (h) median lethal concentration (LC_50_) and evaluation of the long-term effects of BP. Four experimental groups (n = 30) were established, with two for each phase: the control and treatment groups, each with three replicates (n = 10). The fish (n = 120) were randomly assigned to the experimental groups. The LC_50_ and lethal concentration of BP for *L. rohita* were determined using an acute toxicity bioassay for 96-h according to the OECD guidelines [30]. The fish were placed in 50-L glass aquaria containing reconstituted aerated tap water. The physicochemical parameters of the water were maintained during both phases of the experiment according to the protocol described by APHA [5] Table 1. The control group contained only the reconstituted water (no BP). In the treatment group, the fish were exposed to gradually increasing concentrations of BP from 100 µg/L to 1000 µg/L for 96-h. The concentrations used for exposure agreed with those found in several studies that investigated the effects of BP UV filters on different fish species [3], [2], [23]. Half of the water in the aquaria was replaced daily with fresh water at the same BP concentrations to maintain constant concentrations, and no feed was provided during this trial phase. Fish were considered dead if they showed no movement, fish mortality was noted, and dead fish were removed every 24-h to prevent contamination of the test media. At the end of the exposure period, probit analysis was performed based on the cumulative mortality data recorded at different concentrations to determine the 96-h LC_50_ and lethal concentration.

Based on the 96-h LC_50_ value of BP, sublethal toxicity testing was performed by exposing fish to a sublethal dose of BP (1/5th) viz 122.4 µg/L with three replicates for 35 d to observe the histopathological changes in the exposed organs. A separate control group was also maintained. The fish were fed twice daily during the sublethal exposure. The sublethal dose and duration were selected based on a previous study [6].

### 2.3 Histopathological analysis

Histological analysis was conducted according to the method used in previous studies with minor modifications [25], [40], [32]. After the exposure period, the fish were euthanized by hypothermia and dissected to isolate the gills and muscles. The histopathological examination of the organs was performed under a light microscope. The organs were sampled and preserved in 10 % formalin for 10 d, embedded in wax using steel molds, and cut into broad slices (5–6 µm thick) using a rotary microscope. The slices were stained with hematoxylin and eosin. The slices of the isolated organs were examined under a light microscope at a magnification of 40X. The BP-exposed tissues were compared to the control tissues.

### 2.4 Statistical analysis

The Probit analysis method using Minitab software version 19 was employed with a 95 % confidence interval based on the cumulative mortality data recorded at different concentrations to determine the 96-h LC_50_ and lethal concentration. The probit analysis method was used because of its accurate and robust estimation of the LC_50_ and lethal concentration values [18].

## 3 Results

### 3.1 Cumulative mortality and LC_50_ determination

**Fig. 1:**
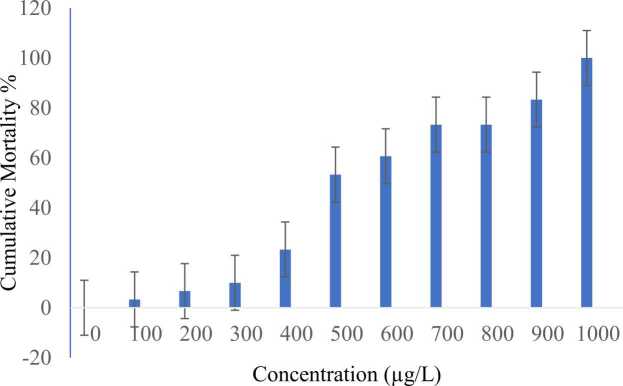
Cumulative mortality percentage of L. rohita exposed to BP (µg/L) for the period of 96 h. The graph represents the overall pattern of the percentage of cumulative mortality of L. rohita at various BP concentrations during 96-h exposure. The blue area represents fish mortality. It showed that fish mortality gradually increases with increasing concentrations of BP. Alt text: Cumulative mortality of L. rohita exposed to BP for 96-h.

**Fig. 2:**
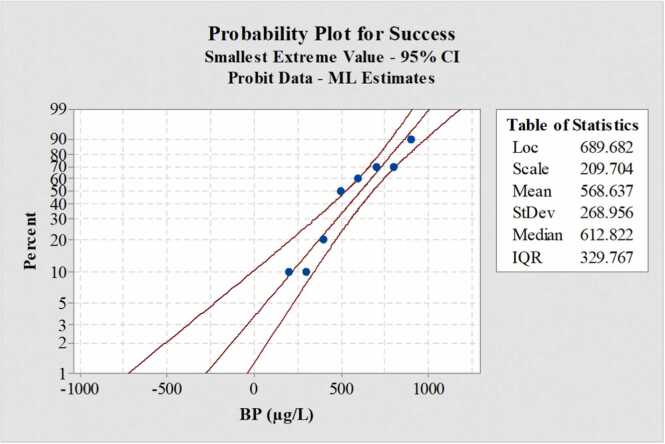
Probability graph of percent mortality of L. rohita against different concentrations of BP (µg/L). The probability graph plots the percent mortality of L. rohita against different BP concentrations to determine the 96-h LC_50_ and lethal concentration. Alt text: Mortality Probability graph of L. rohita during 96-h exposure.

Fish mortality gradually increased with increasing BP concentrations (Fig. 1). The mean LC_50_ concentration was determined as 612.822 ± 37.38 µg/L (95 % CI =683.22–524.18 µg/L) and the lethal concentration was measured as 1009.94 ± 64.44 µg/L (95 % CI = 1184.77–908.59 µg/L). Mortality was not observed in the control group. The 96-h LC_50_ and lethal concentrations of BP are presented in Table 2. A probability graph for the percentage of mortality is shown in (Fig. 2).

**Table 2:** 96-h LC_50_ and lethal concentration determination of BP (µg/L) for L. rohita. The table shows the determination of 96-h LC_50_, lethal concentration, and their 95 % Confidence Interval (CI) with Lower Confidence Level (LCL) and Upper Confidence Level (UCL) of BP to *L. rohita*.

**Fish specie**	**Chemical**	**LC**_**50**_	**95 %CI LCL-UCL**	**Lethal conc.**	**95 %Cl LCL-UCL**	**Chi-Square**	**Df P value**
*L. rohita*	BP	612.822 ± 37.38	683.22–524.18	1009.94 ± 64.44	1184.77–908.59	5.24	9 0.81

### 3.2 Histopathology

**Fig. 3:**
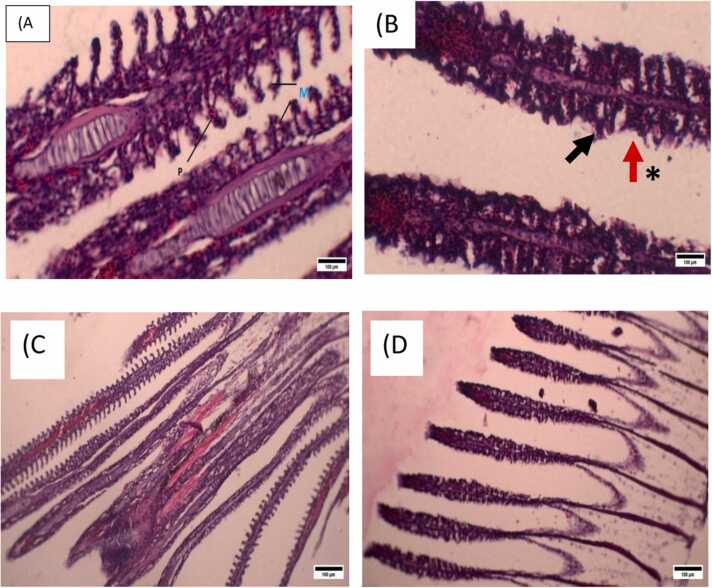
Gills Histology of control and BP-treated groups of L. rohita. Histological images of the gills of control and BP-treated groups of L. rohita. (A) Normal gill's histology shows the mucous cells (M) and pillar cells (P) of the Control group. (B) Histology of the gills treated with BP depicting intraluminal debris (black arrows) and the significant (*) Cellular damage in mucous cells and pillar cells. A&B at 40X. (C) and (D) represents the gills histology at 10X. Alt text: Histological images of the gills of L. rohita.

Different histopathological alterations were observed in the BP-treated gills of *L. rohita*. Photomicrographs of both control and treated groups are shown (Fig. 3). The control group had normal histology, depicting pillar and mucous cells (Fig. 3A) whereas damage in pillar and mucous cells was observed in the treatment group. Moreover, intraluminal debris in the mucous cells of the gills was also observed (Figs. 3B, 3C, and 3D).

**Fig. 4:**
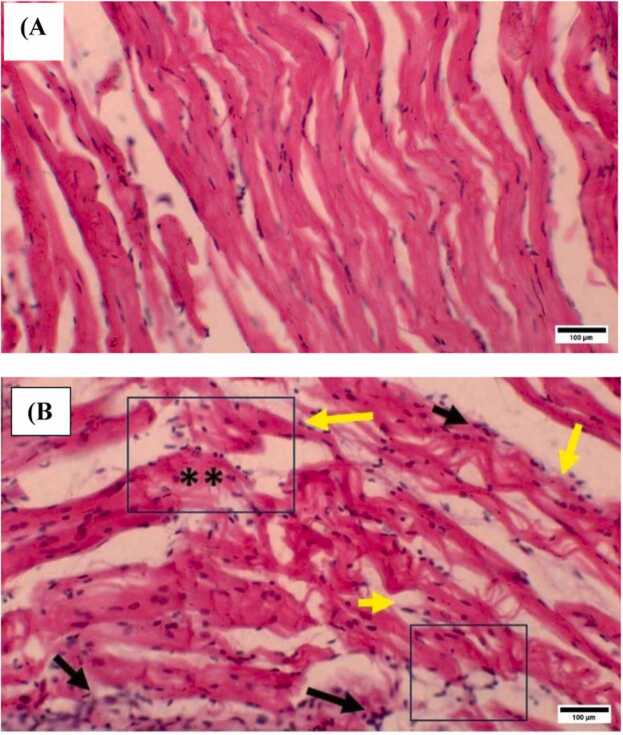
Muscle histology of control and BP-treated group of *L. rohita.* Histopathological images of the muscle of control and BP-exposed groups of L. rohita. (A) Normal histology of muscle of control group. (B) Histology of BP-treated muscle represented significant (**) damages induced by BP, black arrows represent vascular necrosis and intraluminal debris and yellow arrows represent muscle fragmentations of intact structure. (Hematoxylin & Eosin staining, 40X). Alt text: Muscle histology of L. rohita.

In this study, histopathological changes in the muscles of *L. rohita* were also evaluated, and photomicrographs are shown (Fig. 4). No damage was observed in the control group muscle (Fig. 4A), whereas Intraluminal debris was found in the treatment group muscle. BP also fragmented the intact structures and induced vascular necrosis in the exposed muscle (Fig. 4B), confirming the significant damage in treated muscles.

## 4 Discussion

BP is toxic to aquatic organisms because it accumulates in the body due to its lipophilic nature [21]. That is why assessment of the safety and tolerance limits of chemicals is important before their use in industries. For this purpose, determining the lethal concentration of a chemical is important. The term ‘lethal concentration’ refers to the mortality rate of experimental organisms during a specific period. This method has been used to observe the impacts of certain dosages and chemical substances on biological and ecological systems [22]. This study confirms previous findings showing the lethality of BP at elevated concentrations in fish species. The 96-h LC_50_ for *L. rohita* was determined as 612.822 ± 37.38 µg/L (95 % CI =683.22–524.18 µg/L). Our results are consistent with previous studies by Du et al. [13]; Meng et al. [27]; Zhang et al. [47], who studied the lethality of various BP-UV filters. Results of Du et al. [13] showed that 96-h LC_50_ values of BP3 (3.89mgL^−1^) and BP4 (633.00mgL^−1^) for *BrachyDanio rerio* indicated the potential toxicity of BP3 as compared to BP4. Meng et al. [27] reported the 96 h LC_50_ values of BP1 (6.5 µM), BP3 (18.2 µM), and BP8 (4.7 µM) in zebrafish larvae. Similarly, Zhang et al. [47] measured the 96-h LC_50_ of BP and oxybenzone in zebrafish embryos as 9.75 mgL^−1^ and as 4.40 mgL^−1^ respectively, in the absence of UV light, and 4.52 mgL^−1^ and 1.69 mgL^−1^ in the presence of UV light. Results of our study and previous studies confirmed the lethality of BP UV filters in fish. However, neither a standard value was found nor a similar trend was observed for any filter. Du et al. and Zhang et al. reported BP3 as more toxic whereas Meng et al. reported BP8 as more toxic relative to BP1 and BP3, and our LC_50_ toxicity testing value is lower than these previously published values. The difference in the trends shows that the toxicity of BP compounds can vary significantly depending on the fish species and its stage, BP derivatives, exposure conditions, and the presence or absence of UV light.

The sublethal concentration of BP induced different histopathological responses in the gills and muscles of *L. rohita* individuals after 35 d, indicating organ-specific responses in BP-exposed fish. The toxicity of BP UV filters has already been reported in fish species, such as rainbowfish (*Poecilia. reticulata)* [3] and zebrafish [7], [27], [38]. however, little is known about their histopathological effects in fish [7], [25], To address this gap, we focused on histopathology as the main parameter in our study. To the best of our knowledge, the present study is the first to examine the muscle histology of fish exposed to BP. This study observed several histopathological changes in treated gills and muscles. Control group *L. rohita* gills had normal morphology. However, BP caused changes in the morphology of gills. Results have shown the damage to the pillar and mucous cells. In addition, intraluminal debris in the gills mucous cells was also observed. Pillar cells are modified endothelial cells that are unique to fish gills and are present between the lamellar capillaries, facilitating blood flow through the gills for gas exchange [36]. Pillar cell damage indicates an inflammatory response, and such damage disrupts the normal blood flow in the secondary lamellae, leading to blood congestion or an aneurysm development [19]. Aneurysms in the secondary lamellae are often observed in fish exposed to wastewater [33], [37] which is one of the main sources of BP. Mucous cells are large and ovoid, with cytoplasm containing mucus secretory granules that produce mucous that acts as a lubricant and barrier. These are on the lamellar base and edges [36]. Fish gills respond to excess environmental contaminants by increasing mucous production as a defense mechanism, resulting in cellular damage and debris releases in the gill lumen [9]. These damages and accumulation of intraluminal debris affect the basic physiological processes of fish, such as gas exchange, osmoregulation, and antioxidant defense mechanisms, collectively impairing the fish's respiratory efficiency and increasing susceptibility to low oxygen or environmental stressors affecting the overall fish health. These disturbances can be observed much earlier than external appearance or behavioral changes in fish [45]. Almost similar histopathological alterations affecting the morphology of the gills were observed by some other researchers who studied the effects of the BP3 UV filter on the gills histopathology of zebrafish and rainbowfish, such as progressive and regressive changes, circulatory disorders, inflammatory responses, and destruction of secondary lamellae [2], [24], lamellar structure disorganization, gill filament fusion, and red blood cells (RBCs) inflammation that obstructed blood material in the primary lamella (PL), complete fusion of the secondary lamella (SL), and vacuolization of the PL [40]. These histopathological changes in gill structure observed across different fish species exposed to BP and its derivatives are consistent with the broader literature on the impact of environmental pollutants on fish gills.

*L. rohita* exposed to sublethal concentration of BP exhibited histopathological changes in the muscle. Results reported the fragmentation of intact structures, intraluminal debris, and vascular necrosis in the muscles. Vascular necrosis results from the obstruction of the blood flow in vessels that causes insufficient blood supply to cells, resulting in cell death and fragmentation of the muscle fibers. The presence of intraluminal debris can be linked to fragmented muscle fibers, indicating an inflammatory response leading to cellular debris [35]**.** Muscles were found to be less affected than the gills because they are not in direct contact with the environmental toxicants. To the best of our knowledge, we could not find any previous study that evaluated the toxic effects of BP on fish muscle histopathology. However, researchers who studied the effects of other toxicants on fish species reported similar results. Kaur et al. [20] and Shahid et al. [35] evaluated the toxicity of heavy metals and industrial pollution in *L. rohita* and catfish (*Ictalurus punctatus)* muscle tissues and found the edema, necrosis, muscle atrophy, and muscle bundle shortening. In addition, degeneration of muscle myofibrils, necrosis, splitting of muscle fibers, thickening of muscle bundles, and dislocated striated muscles were observed in the muscle tissues of African catfish (*Clarias garepinus*) exposed to silver nanoparticles [34]. Nile tilapia (*Oreochromis niloticus)* muscles indicated epithelial layer and muscle fibers degeneration, necrosis, and edema in the hypodermal layer due to heavy metals accumulation [43]. These studies demonstrated the toxic effects of environmental toxicants on fish muscle tissues. The results of our study confirmed the hypothesis that BP induces histopathological changes in the muscles and gills of *L. rohita*, highlighting the environmental risks associated with this chemical*.* These results agree with previous studies and indicate that these changes can impair the gills and muscle-related physiological functions and affect the overall health of the fish population.

## 5 Conclusion

Our study evaluated the toxic effects of BP on the gills and muscles of *L. rohita* and reported that long-term exposure induced histopathological alterations in these exposed organs. These results are consistent with previous studies demonstrating the toxic effects of BP UV filters on fish and other aquatic organisms. These findings and previously reported results established that exposure to BP UV filters disrupts the normal physiological functions of fish bodies, causing reduced growth, reproduction, and survival, ultimately affecting the *L. rohita* population. Strict regulatory control on the release of BP into waterbodies is necessary. BP filters do not have any permissible limits set by any global environmental organization. This omission highlights the huge regulatory gap for this specific chemical. Therefore, it is recommended that the Environmental Protection Agency (EPA) establish specific permissible limits for BP UV filters in drinking water. Effective implementation of wastewater treatment technologies can also play an important role in reducing the prevalence of BP in aquatic environments. Advanced treatment technologies such as advanced oxidation processes, reverse osmosis, and nanofiltration are found to be more effective in removing BP from wastewater, these technologies must be scaled up to large WWTPs and industrial purposes. Future toxicological studies should focus on developing ecotoxicological models that will help to access the broader implication of BP on aquatic ecosystems.

## Author Statement

“I, the Corresponding Author, declare that this manuscript is original, has not been published before, and is not currently being considered for publication elsewhere.

I can confirm that the manuscript has been read and approved by all named authors and that there are no other persons who satisfied the criteria for authorship but are not listed. I further confirm that the order of authors listed in the manuscript has been approved by all of us.

I understand that the Corresponding Author is the sole contact for the Editorial process and is responsible for communicating with the other authors about progress, submissions of revisions, and final approval of proofs.

## CRediT authorship contribution statement

**Maham Fatima:** Visualization. **Amna Rasheed:** Resources. **Urwah Sheikh:** Formal analysis. **Sajid Abdullah:** Supervision, Methodology, Investigation. **Mina Jamil:** Writing – review & editing, Methodology. **Maham Riaz:** Writing – original draft. **Nimra Umer:** Data curation. **Kaynat Aslam:** Data curation.

## Declaration of Competing Interest

The authors declare that they have no known competing financial interests or personal relationships that could have appeared to influence the work reported in this paper.

## Data Availability

Data will be made available on request.
